# Multiple roles of Bet v 1 ligands in allergen stabilization and modulation of endosomal protease activity

**DOI:** 10.1111/all.13948

**Published:** 2019-10-08

**Authors:** Wai Tuck Soh, Lorenz Aglas, Geoffrey A. Mueller, Stefanie Gilles, Richard Weiss, Sandra Scheiblhofer, Sara Huber, Tamara Scheidt, Peter M. Thompson, Peter Briza, Robert E. London, Claudia Traidl‐Hoffmann, Chiara Cabrele, Hans Brandstetter, Fatima Ferreira

**Affiliations:** ^1^ Department of Biosciences University of Salzburg Salzburg Austria; ^2^ Department of Health and Human Services, Genome Integrity and Structural Biology Laboratory National Institute of Environmental Health Sciences, National Institutes of Health Research Triangle Park North Carolina; ^3^ Institute of Environmental Medicine UNIKA‐T, Technical University Munich and Helmholtz Zentrum München Augsburg Germany; ^4^ Christine‐Kühne‐Center for Allergy Research and Education (CK CARE) Davos Switzerland

**Keywords:** allergenicity, birch pollen extract, E_1_ phytoprostanes, ligand interaction, lysosomal protease inhibition

## Abstract

**Background:**

Over 100 million people worldwide suffer from birch pollen allergy. Bet v 1 has been identified as the major birch pollen allergen. However, the molecular mechanisms of birch allergic sensitization, including the roles of Bet v 1 and other components of the birch pollen extract, remain incompletely understood. Here, we examined how known birch pollen–derived molecules influence the endolysosomal processing of Bet v 1, thereby shaping its allergenicity.

**Methods:**

We analyzed the biochemical and immunological interaction of ligands with Bet v 1. We then investigated the proteolytic processing of Bet v 1 by endosomal extracts in the presence and absence of ligands, followed by a detailed kinetic analysis of Bet v 1 processing by individual endolysosomal proteases as well as the T‐cell epitope presentation in BMDCs.

**Results:**

We identified E_1_ phytoprostanes as novel Bet v 1 ligands. Pollen‐derived ligands enhanced the proteolytic resistance of Bet v 1, affecting degradation kinetics and preferential cleavage sites of the endolysosomal proteases cathepsin S and legumain. E_1_ phytoprostanes exhibited a dual role by stabilizing Bet v 1 and inhibiting cathepsin protease activity.

**Conclusion:**

Bet v 1 can serve as a transporter of pollen‐derived, bioactive compounds. When carried to the endolysosome, such compounds can modulate the proteolytic activity, including its processing by cysteine cathepsins. We unveil a paradigm shift from an allergen‐centered view to a more systemic view that includes the host endolysosomal enzymes.

## Graphical abstract

The newly identified birch pollen‐derived ligands, Phytoprostane E1 (PPE_1_), interacts with Bet v 1 affecting its stability and proteolytic processing. Ubiquitous plant phytoprostanes, PPE_1_, covalently inhibit lysosomal cysteine cathepsins, with multiple consequences including effects on antigen processing. PPE_1_ affected the presentation of Bet v 1 T‐cell epitope in BMDC to T‐cell.

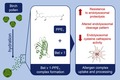

**Abbreviations**
AMC: 7‐amino‐4‐methylcoumarin
ANS: 8‐anilinonaphthalene‐1‐sulfonic acid
BMDC: bone marrow–derived dendritic cell
BPE: birch pollen extract
CD: circular dichroism
CMK: chloromethyketones
DC: dendritic cell
DOC: sodium deoxycholate
DTT: dithiothreitol
EDTA: ethylenediaminetetraacetic acid
FACS: fluorescence‐activated cell sorting
FcεRI: high‐affinity IgE receptor
FTIR: Fourier transform infrared spectroscopy
K_d_: equilibrium dissociation constant
Kdo_2_: Kdo_2_‐Lipid A
LPS: lipopolysaccharide
LTA: lipoteichoic acid
moDCs: monocyte‐derived dendritic cells
MW: molecular weight
NLR: NOD‐like receptor
NMR: nuclear magnetic resonance
PPA_1_: PPB_1_, PPE_1_, PPF_1_, phytoprostane A_1_, B_1_, E_1_, and F_1_
PPAR‐γ: nuclear peroxisome proliferator–activated receptor γ
Q3OS: quercetin 3‐O‐sophoroside
SAW: surface acoustic wave
SDS‐PAGE: sodium dodecyl sulfate‐polyacrylamide gel electrophoresis
TCEP: tris(2‐carboxyethyl)phosphine
TLR: Toll‐like receptor

## 1 INTRODUCTION

An allergic response is a two‐step process, involving an initial sensitization step characterized by a pronounced Th2 polarization and followed by an acute antibody recognition step.^1^ While the latter can be triggered by isolated allergen molecules alone, such as the primary birch pollen allergen Bet_v_1, the initial sensitization process is more complex. We recently found that, in the case of birch (*Betula verrucosa)* pollen allergy, Th2 polarization is not driven by its major allergen Bet_v_1.^2^ This observation makes the role of Bet_v_1 as a major allergen even more intriguing.^3^, ^4^ In this context, Bet_v_1’s ability to function as a carrier or storage protein for a wide variety of natural hydrophobic ligands has been discussed.^5^ Indeed, several allergens have been investigated concerning their lipid‐binding properties as a determinant of allergenicity.^6^

Three major groups of compounds have been proposed to interact or cooperate with Bet_v_1, two of which are pollen‐derived: (a) flavonoids, (b) phytohormones, and (c) microbe‐derived Toll‐like receptor (TLR) agonists. In a previous study, the glycosylated flavonoid quercetin 3‐*O*‐sophoroside (Q3OS) was found to co‐purify with Bet_v_1 from pollen and therefore reported as a physiological Bet_v_1 ligand.^7^ Phytohormones, including phytoprostanes and brassinosteroids, are low‐molecular‐weight compounds present in pollen extract. While the ability of Bet_v_1 to bind brassinosteroids has been demonstrated,^8^ physical interactions with Bet_v_1 have not yet been reported for phytoprostanes. Phytoprostanes like E_1_ (PPE_1_) are functionally related to mammalian prostaglandins and possess Th2‐skewing activity, making them of potential interest as a sensitization mechanism.^9^ Other ligands of interest include deoxycholate (DOC), a secondary bile acid generated as a microbial metabolic byproduct that is structurally similar to brassinosteroids^10^ and serves as an established model ligand for Bet_v_1.^10^, ^11^ In addition, immunomodulatory microbial compounds (such as the TLR2 and NLRP6 agonist lipoteichoic acid, LTA, and the endotoxin lipopolysaccharide, LPS) have been proposed to interact with Bet_v_1.^6^, ^12^, ^13^, ^14^, ^15^

Bet_v_1 ligands have been proposed either to exhibit direct immunomodulatory functions^16^ or to stabilize the Bet_v_1 conformation indirectly, which could change its immunogenicity and allergenicity by influencing its processing in the endolysosome.^17^, ^18^ Among endolysosomal proteases, the large family of cathepsins, most of which are cysteine proteases belonging to the papain family, plays an important role in proteolytic activity.^19^ Only a few other proteases have been shown to be relevant in antigen processing, including the cysteine protease legumain.^20^ As such, the endosomal degradation of Bet_v_1 can be modeled by microsomal extracts and reproduced using purified extracts, particularly cathepsin S and legumain.^21^

In this study, we biochemically and immunologically dissected the interactions of recombinant Bet_v_1.0101 (termed Bet_v_1 in the following), the most abundant isoform of Bet_v_1 present at approximately 50%‐70%,^22^ with several ligands, including Q3OS, PPE_1_, and DOC. Remarkably, PPE_1_ was not only retained by Bet_v_1, but also inhibited the cysteine cathepsins in the endolysosome. We discuss the implications of these new findings for our understanding of pollen‐derived allergy.

## 2 MATERIALS AND METHODS

A detailed description of the methods is provided in the Appendix [Link], [Link].

### 2.1 Expression, purification, and physicochemical characterization of recombinant Bet_v_1

Production of recombinant Bet_v_1.0101 and monitoring of endotoxin contamination (<0.3 ng/mL) were performed as previously described.^3^, ^11^

### 2.2 Investigated compounds and Bet_v_1 ligands

DOC, 8‐anilinonaphthalene‐1‐sulfonic acid (ANS), naringenin, LTA from *Staphylococcus aureus*, and LPS from *Escherichia coli* O111:B4 were purchased from Sigma‐Aldrich, Inc; Kdo_2_‐Lipid A (Kdo_2_) from Adipogen, Inc or Avanti Polar Lipids, Inc; and quercetin 3‐*O*‐sophoroside (Q3OS) from Haihang Industry Co., Ltd. PPE_1_, B_1_‐phytoprostanes (PPB_1_), F_1_‐phytoprostanes (PPF_1_), and an isomeric mixture consisting of B_1_‐, E_1_‐, and F_1_‐phytoprostanes (PP_mix_) were produced by autoxidation of α‐linolenic acid, as described elsewhere.^23^ Type I or/and type II phytoprostanes were used, as indicated in Figure 4C. Unless otherwise stated, Bet_v_1 was mixed with each of the six ligands in a 1:10 molar ratio and incubated either overnight at 4°C or for 2 hours at room temperature. A_1_‐phytoprostanes (PPA_1_) were purchased from Cayman Chemicals and dried and dissolved in DMSO.

### 2.3 Protein‐ligand interaction

Surface acoustic wave (SAW) technology and NMR spectroscopy were used to observe the interaction of Bet_v_1 with the selected compounds, including determination of the dissociation constant (*K*_d_). The influence of ligand binding on the secondary structure elements and the thermal stability of Bet_v_1 was monitored using circular dichroism (CD, JASCO J‐815 spectropolarimeter, Jasco) and Fourier transform infrared (FTIR) spectroscopy (Tensor II FTIR system, Bruker Optics Inc). A detailed description of these methods is available (Appendix [Link], [Link]).

### 2.4 Immunological assays

The ability of ligand‐loaded Bet_v_1 to induce IgE‐antigen cross‐linking and basophil degranulation was assessed by mediator‐release assays using rat basophil (RBL‐2H3) cells, transfected with the human high‐affinity IgE receptor (FcεRI), as previously described.^2^, ^24^ In vitro uptake of labeled Bet_v_1 was performed using CD11c^+^ murine bone marrow–derived dendritic cells (BMDCs). The maturation of human monocyte‐derived dendritic cells (moDCs) was analyzed as previously described.^2^ T‐cell proliferation assays using CD4^+^ T‐cell hybridomas were performed as previously described.^17^ A detailed description of the in vitro assays is available (Appendix [Link], [Link]).

### 2.5 In vitro simulation of endolysosomal degradation using microsomes and individual endolysosomal proteases

The endolysosomal degradation assay was performed with ligand‐bound (either DOC, PPE_1_, or Q3OS in 10× molar excess) and Bet_v_1 without ligands (apo‐Bet_v_1) as previously described.^21^ Recombinant human cathepsin S and human legumain were used in proteolytic degradation assays. Experimental details are described in the Appendix [Link], [Link].

### 2.6 Enzymatic activity assays

To evaluate the influence of Bet_v_1 ligands on cathepsin S and legumain activities, 10 nmol/L of protease was incubated with 100 µmol/L of ligand (unless otherwise stated) and 50 µmol/L of fluorogenic substrate in digestion buffer (0.1 mol/L sodium acetate pH 5.0, 0.1 mol/L sodium chloride, 5 mmol/L EDTA, and 2 mmol/L DTT), as described in the Appendix [Link], [Link]. The effect of birch pollen extract (BPE) (20‐200 µg/mL) on the cathepsin S and legumain activities was assessed in parallel. The inhibitory effect of PPE_1_ was assessed by replacing DTT with 0.5 mmol/L TCEP. Activities of recombinant rat cathepsin B (provided by Dr Lukas Mach) and papain (Merck) at 10 nmol/L were assayed using Z‐FR‐AMC (Bachem) as a fluorogenic substrate.

## 3 RESULTS

### 3.1 Bet_v_1 interacts with high affinity with pollen‐derived PPE_1_ and Q3OS and with the brassinosteroid‐like compound DOC, but not with LTA or LPS

To assess the interactions between Bet_v_1 and Q3OS, DOC, PPE_1_, LTA, or LPS, we determined the dissociation constants (*K*_d_) using SAW binding assays (Table 1, Figure S1), a more quantitative approach than previously described qualitative assays.^11^, ^25^ In addition, the LPS‐substructure Kdo_2_‐Lipid A (Kdo_2_) was used for binding studies, due to its more homogenous structure but similar immune stimulatory activity when compared to native LPS.

**Table 1:** Binding affinity (*K*_d_) of Bet_v_1 to the selected compounds as determined by SAW interaction studies and binding confirmation by NMR spectroscopy

	Compound	MW [Da]	*K*_d_ [µmol/L]	SD [µmol/L]	NMR [µmol/L]
Pollen‐derived compounds	Q3OS	626.5	1.5	±0.1	[7]
	PP_mix_		1.2	±0.1	n.d.
	PPB_1_	308.4	1.0	±0.4	n.d.
	PPF_1_	328.4	2.4	±0.5	n.d.
	PPE_1_	356.5	0.5	±0.1	0.1‐1
	PPA_1_	308.4	n.d.	n.d.	0.1‐1
Model compounds mimicking essential binding groups	DOC	414.6	58.8	±24.3	[11]
	ANS	299.34	32.7	±0.3	[11]
Bacteria‐derived compounds	LTA	4000‐8000	199.8	±55.7	No significant interactions
	LPS	10 000‐20 000	185.0	±123.1	No significant interactions
	Kdo_2_	2306.8	379.8	±62.8	No significant interactions

As a reference ligand, the binding of ANS to Bet_v_1 was determined (*K*_d_ of 32.7 µmol/L) which is similar to previously published *K*_d_ values (18.5 µmol/L).^26^ The two pollen‐derived components, Q3OS and PPE_1_, exhibited high binding affinities with *K*_d_ = 1.5 and 0.5 µmol/L, respectively. The bacterial TLR agonists, LTA (199.8 µmol/L) and LPS (185.0 µmol/L), and the model substances, DOC (58.8 µmol/L) and Kdo_2_ (379.8 µmol/L), demonstrated higher *K*_d_ values, indicating lower binding affinities. For the phytoprostane derivatives, PPB_1_ and PPF_1_, as well as for a physiologically relevant isomeric mixture consisting of B_1_‐, E_1_‐, and F_1_‐phytoprostanes (PP_mix_), we observed dissociation constants of 1.0, 2.4, and 1.2 µmol/L, respectively.

To validate the interactions determined by SAW, we used NMR spectroscopy to test the specific binding of PPE_1_, LTA, LPS, and Kdo_2_ to Bet_v_1 (Table 1, Figure S2). Substantial differences between the^1^H‐15N HSQC spectra of 15N‐labeled Bet_v_1 in the absence and presence of PPE_1_ confirmed that the allergen specifically binds PPE_1_. The *K*_d_ was consistent with a low to sub‐µmol/Laffinity, but intermediate exchange and a poor signal‐to‐noise ratio prevented direct measurement. The commercially available PPA_1_ was used as a substitute for PPE_1_ to identify the phytoprostane binding site(s). No significant interactions were observed for LTA, LPS, or Kdo_2_, indicating that these bacterial compounds do not specifically bind to Bet_v_1, consistent with LPS pull‐down assays using Bet_v_1 and biotinylated LPS immobilized on Strep‐Tactin Sepharose beads (Figure S3).

Moreover, using CD and FTIR spectroscopy we observed an increased melting point (*T*_m_) of approximately 4°C and nearly 7°C for Bet_v_1 bound to DOC and PPE_1_, respectively (Table 2). Bet_v_1Binding of DOC, Q3OS or PPE_1_ to Bet_v_1 did not significantly alter its secondary structure content (Figure S4).

**Table 2:** Influence of ligand interaction on thermal stability of Bet_v_1 (values in °C)

Ligand	*T*_m_ CD	SD CD	*T*_m_ FTIR	SD FTIR	ΔCD	ΔFTIR
‐	63.68	±0.06	63.38	±2.24		
Q3OS	64.04	±0.10	65.26	±1.77	+0.36	+1.88
DOC	67.44	±0.58	66.6	±4.36	+3.81	+3.22
PPE1	70.62	±0.15	69.31	±0.05	+6.94	+5.93

Abbreviations: CD, circular dichroism; FTIR, Fourier transform infrared spectroscopy; *T*_m_, melting point; SD, Standard deviation.

### 3.2 Ligand binding to Bet_v_1 does not affect basophil degranulation or the activation of dendritic cells

We next set out to test for effects on Bet_v_1‐complexes on different stages of the allergic immune response. Antigen uptake was assessed by uptake of pHrodo™ Red‐labeled Bet_v_1, with or without ligands (Figure S5A), and subsequent FACS analysis. Sensitizing potential was assessed on the level of dendritic cells by flow cytometric analysis of maturation marker expression and by determination of Th polarization‐associated cytokines in cell culture supernatants (Figure S5B and C). IgE cross‐linking by Bet_v_1‐complexes was assessed by RBL assay (Figure S6). None of the above described readouts was influenced by the presence of plant‐derived Bet_v_1 ligands (Q3OS, PPE_1_, and DOC).

### 3.3 Ligand interactions with Bet_v_1 influence its lysosomal processing

**Figure 1:**
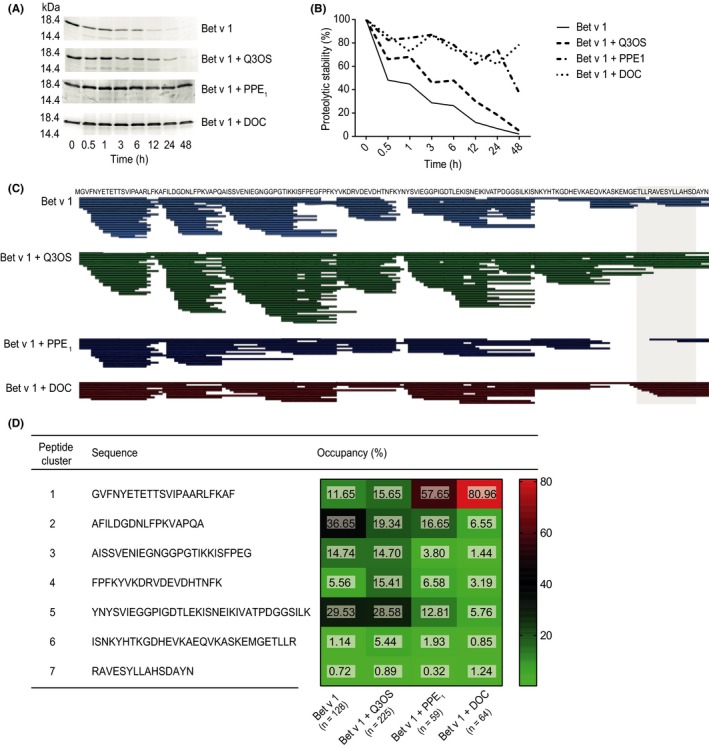
Ligand interaction alters the proteolytic susceptibility of Bet_v_1. A, SDS‐PAGE analysis of in vitro endolysosomal degradation of Bet_v_1 with and without ligand recorded at different time points from 0 to 48 h and B, densitometric analysis thereof, interpreted with Image Lab 4.0.1 Software (Bio‐Rad). C, Generated peptide clusters obtained after 12 h of proteolytic degradation analyzed by mass spectrometry. D, The peptide sequences were grouped into seven degradation clusters with their relative abundance, as derived from MS intensities. The number of unique peptide sequences is shown in brackets

Given the relevance of conformational stability and proteolytic resistance for MHCII presentation,^27^ we prepared endosomal extracts to assess the resistance of Bet_v_1 in complex with the model ligands toward endolysosomal proteases over 48 hours. Densitometric analysis of SDS‐PAGE (Figure 1A and B) revealed an enhanced proteolytic stability of Bet_v_1 in the presence of PPE_1_ and DOC. By contrast, Q3OS had only a weakly stabilizing effect over the first 12 hours. This observation correlated with our thermal stability data.

As the lysosomal resistance of allergens correlates with the quality and quantity of the ensuing immune response,^17^ we analyzed the peptides generated after 12‐hours incubation with endolysosomal proteases (Figure 1C). The binding of Q3OS resulted in a 2‐fold higher diversity of peptides within the different peptide clusters than with the apo form of Bet_v_1, whereas the resulting Bet_v_1 peptide diversity was reduced upon binding of PPE_1_ and DOC (to 53.9% and 69.7%, respectively). In a semi‐quantitative approach, the generated peptides were grouped into seven main core clusters with their relative abundances shown (Figure 1D). The rate of core peptide production and/or elimination was affected by the presence of ligands. In the presence of PPE_1_ or DOC, Bet_v_1 processing preferentially accumulated the two N‐terminal cluster peptides. Bet_v_1 in complex with Q3OS or DOC showed an altered pattern of proteolytic processing, which resulted in a more efficient generation of the immunodominant T‐cell epitope, as indicated by the number of identified peptides (gray box in Figure 1C). Bet_v_1Together, these data show that both the quantity and the quality of the peptide pool available for MHCII presentation are affected by the ligands.

### 3.4 Modeling the microsomal processing of Bet_v_1 by cathepsin S and legumain reveals the mechanistic basis of attenuated degradation

Since an endosomal extract is a complex mixture of various hydrolases, we aimed to break down the complexity of the assay by identifying key proteases of the microsomal extracts and further analyzing the influence of ligand binding to Bet_v_1 on their processing capability. Based on previously described enzymatic data,^21^, ^28^ we tested the microsomal fraction for enzymatic activity toward substrates of cathepsin and legumain, two prominent endolysosomal cysteine protease families with complementary substrate preferences and orthogonal catalytic mechanisms.^29^ Consistent with the literature,^30^ we detected both cathepsin‐like and legumain‐like enzymatic activities in microsomal extracts, and these activities were specifically inhibited by cathepsin S/B and legumain inhibitors (Figure S7).

Consequently, we tested whether cathepsin S or legumain qualitatively reproduced the endolysosomal degradation kinetics of apo and ligand‐bound Bet_v_1. Indeed, processing by the individual proteases was strongly retarded by DOC, and, in the case of cathepsin S, also by PPE_1_. Other reported Bet_v_1 ligands^11^ had either a minor (Naringenin) or no detectable (PPB_1_, ANS) effect on its proteolytic resistance. SDS, which also binds Bet_v_1,^31^ significantly accelerated its degradation by both proteases (Figure 3A and B). By contrast, SDS reduced the cleavage of fluorogenic substrates by cathepsin S (Figure 3A). These observations can be reconciled by assuming that the binding of SDS to Bet_v_1 exposes additional vulnerable sites to the protease.

**Figure 2:**
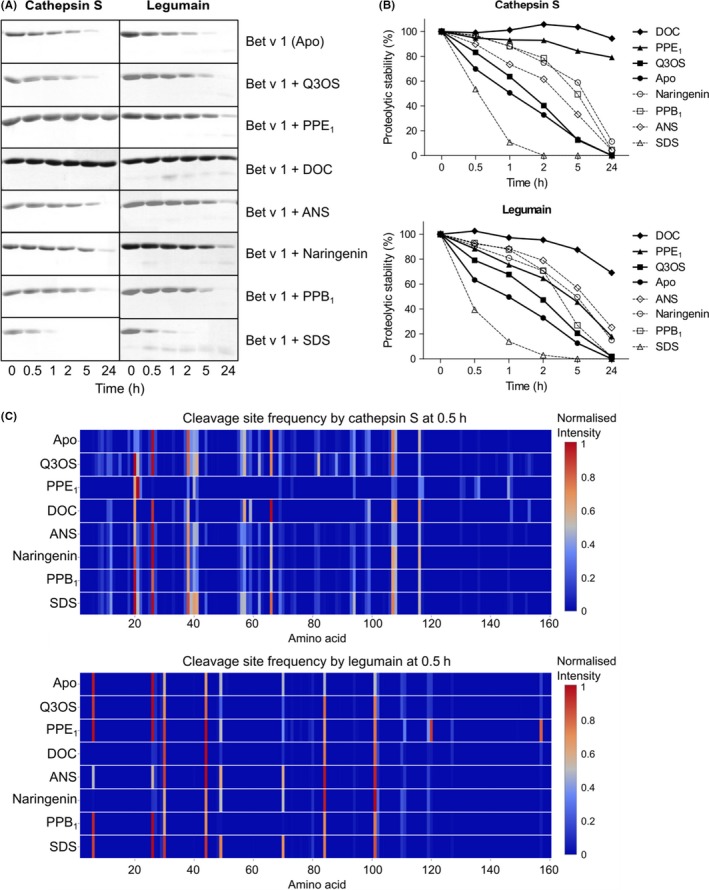
Effect of ligands on Bet_v_1 degradation in vitro. A, Bet_v_1 degradation assay by cathepsin S and legumain in the absence or presence of various ligands. The degradation profile was analyzed by Coomassie Blue‐stained SDS‐PAGE and B, densitometric analysis. C, Bet_v_1 cleavage site frequency analyses of the degradation assay in (A). The analyses were based on the relative abundance of peptides measured by mass spectrometry, and the peptide intensity was normalized to the most abundant peptide found for the respective ligand. This is not a direct representation of the available cleavage sites, but rather emphasizes the varying kinetic accessibility of individual sites for one given ligand. The peptide profiles are presented in Figure S8

The majority of the peptide clusters were generated using cathepsin S alone; however, several cleavage sites after asparagine were only reproduced using legumain, as no other known protease exhibits an asparaginyl‐peptidase activity,^32^ particularly relevant for the production of C‐terminal peptide clusters (Figure 1C, Figure S8). To understand how the pattern and the kinetics of Bet_v_1 processing were affected by the presence of ligands, we analyzed the relative abundance of the resulting peptides. The presence of ligands mostly affected the frequency of cleavages at certain sites within Bet_v_1, but rarely generated new cleavage sites not present in the apo form. PPE_1_ induced prominent changes in relative preference of the Bet_v_1 cleavage sites. Although other ligands affected the cleavage pattern as well, PPE_1_ was used to illustrate the effect of ligand binding on the generation of cleavage sites: Upon incubation with cathepsin S, preferential cleavage was observed after Phe20, Lys21, and in the C‐terminal region; upon incubation with legumain, cleavage frequency after Asn120 and Asp157 strongly increased (Figure 2C). Overall, this analysis shows that the relative abundance of peptides available for MHC presentation is strongly affected by the presence of ligands.

### 3.5 Birch pollen extract reduces cathepsin activity in a dose‐dependent manner

**Figure 3:**
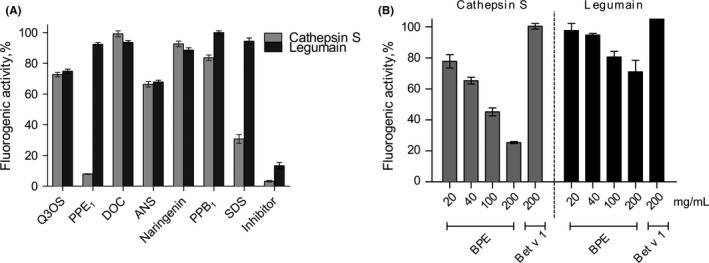
Effect of ligands and birch pollen extract (BPE) on cathepsin S and legumain activities. A, Effect of ligands on fluorogenic activity of lysosomal proteases. B, Effect of BPE on lysosomal protease activity. BPE was incubated with the respective protease, and the fluorogenic activity was measured after 15 min. Recombinant Bet_v_1 was used as control for a possible substrate competition effect. The percent fluorogenic activity was calculated over buffer control. Error bars indicate standard deviations

We wondered whether the observed (de)stabilizing effects of the ligands were caused exclusively by the interaction with Bet_v_1. Therefore, we tested whether the ligands affected protease activity toward small peptidic substrates. Surprisingly, PPE_1_ specifically inhibited cathepsin S, but not legumain (Figure 3A).

Since approximately 0.5 µg of PPE_1_ is present in 1 mg of birch pollen–extracted protein,^9^ we can expect about 150 pmol PPE_1_ in 100 µg of pollen‐extracted protein per mL, that is, 150 nM PPE_1_, in agreement with the reported concentration range.^33^ Although the extraction will come with significant losses, and only type II of PPE_1_ is an active inhibitor, we hypothesized that BPE at corresponding concentrations should also attenuate proteolytic activity. Therefore, we investigated the influence of BPE on cathepsin S and legumain activity (Figure 3B). In contrast to the marginal effects on legumain activity, a dose‐dependent inhibition of cathepsin S was observed. Bet_v_1 at the highest concentration (200 µg/mL) was used to exclude possible substrate competition effects. These data suggest that the BPE‐mediated cathepsin S inhibition may be partially caused by PPE_1_.

### 3.6 PPE_1_ inhibits lysosomal cathepsins by blocking their catalytic cysteine

**Figure 4:**
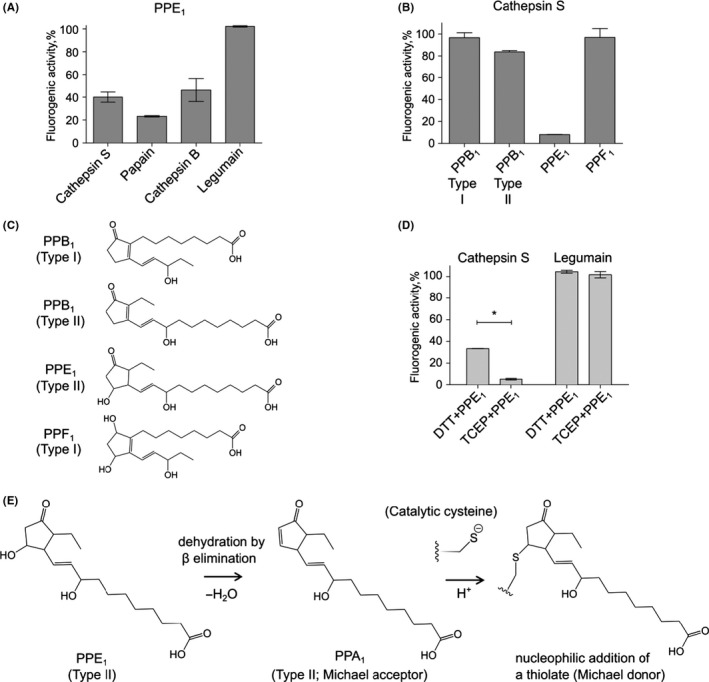
Inhibition mechanism of PPE_1_. A, PPE_1_ inhibits papain‐like cysteine proteases, but not legumain. Papain‐like cysteine proteases (rat cathepsin B, cathepsin S, and papain) and legumain were incubated with PPE_1_ (5 µmol/L), and fluorogenic activities were recorded after 15 min. B, Effect of phytohormones (0.1 mmol/L) structurally related to PPE_1_ on cathepsin S activity. Fluorogenic activity was recorded after 15 min. C, Chemical structure of phytohormones used in (B). D, Effect of reducing agents on PPE_1_ inhibition of cathepsin S and legumain. The ability of proteases to cleave the fluorogenic substrates with and without PPE_1_ (5 µmol/L) in the presence of DTT and TCEP. Fluorogenic substrates used for cathepsin S and legumain were Z‐VVR‐AMC and Z‐AAN‐AMC, respectively. Error bars indicate standard deviations. Asterisk indicates statistical significance with *P* < 0.05. E, Proposed mechanism of cathepsin S inhibition by PPE_1_. PPE_1_ undergoes spontaneous dehydration by β‐elimination, resulting in PPA_1_.^43^ This reaction does not occur with PPB_1_, which lacks a hydroxyl group in the ring, and is disfavored in PPF_1_ due to the missing ketone group. The resulting PPA_1_ is an electrophile (Michael acceptor) and can be readily attacked by the nucleophilic cysteine of cathepsin S (Michael donor) at the β carbon to form a covalent adduct,^48^ thus inhibiting cathepsin S activity

To further investigate the mechanism of PPE_1_‐mediated inhibition, we analyzed other proteases and found PPE_1_‐mediated inhibition of the papain‐like protease family, such as cysteine cathepsins. By contrast, legumain, which belongs to a different protease class, was not inhibited (Figure 4A). Importantly, the structurally similar PPB_1_ and PPF_1_ did not inhibit cathepsin S activity (Figure 4B and C). We wanted to examine whether PPE_1_ exerts its effect by reacting with the nucleophilic cysteine thiol in the active site, a characteristic for this protease class. Therefore, we compared the effect of two reducing agents, (a) the thiol‐containing DTT and (b) tris(2‐carboxyethyl)phosphine (TCEP), which lacks any thiol groups. Cathepsin S activity was completely abolished by PPE_1_ in the presence of TCEP, but not in the presence of DTT (Figure 4D, Figure S9). This differential effect can be understood by DTT thiols competing for the reactive site on the PPE_1_ inhibitor. By contrast, no inhibitory effect on legumain by PPE_1_ was found. In the absence of PPE_1_, we found high cathepsin S activity toward a fluorogenic substrate in the presence of both TCEP and DTT (Figure 4D, Figure S9). The slightly stronger activity‐enhancing effect of TCEP vs DTT is due to its stronger reducing capacity at acidic pH.^34^

### 3.7 PPE_1_ and DOC affect Bet_v_1 processing and presentation in DCs

**Figure 5:**
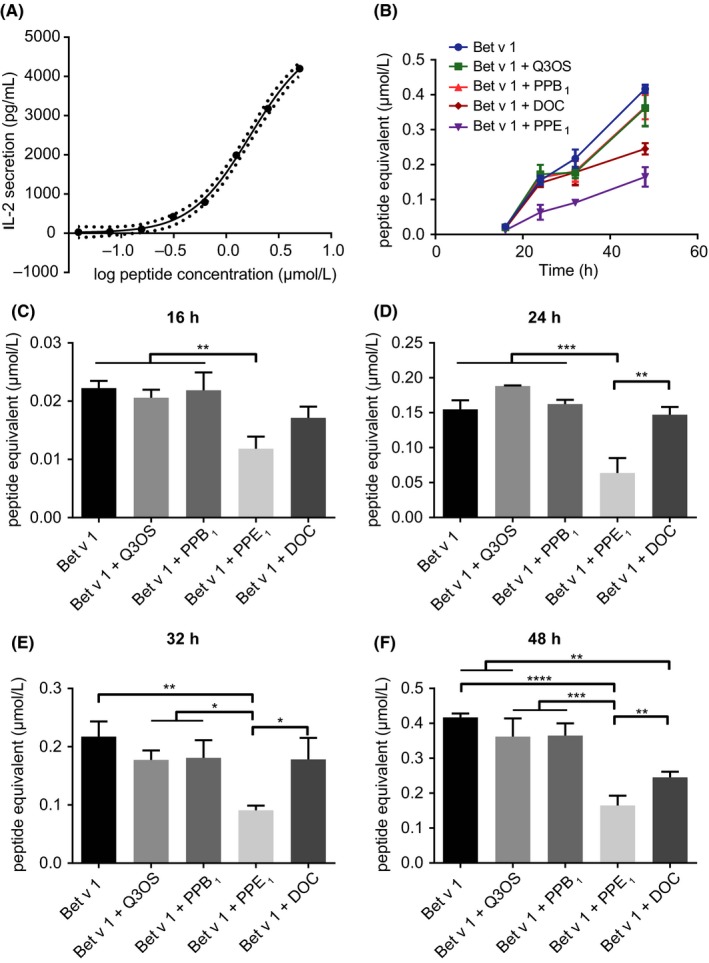
Effect of ligand binding on the Bet_v_1‐specific presentation of BMDCs to CD4^+^ T cells. A, Dose‐response curve relating the IL‐2 secretion of T‐cell hybridoma cells (in pg/mL) to the logarithmic concentration of the corresponding immune‐dominant peptide (Thr142‐Ala153) upon presentation by BMDCs. B, kinetics of Bet_v_1 T‐cell epitope presentation by BMDCs from 16 to 48 h. C‐F, comparison of the presented Bet_v_1 T‐cell epitope in dependency of involved ligand at each individual time point (16, 24, 32, and 48 h). P‐values were calculated with one‐way ANOVA and a Tukey’s multiple comparisons test. All statistical calculations were performed using GraphPad Prism 7 software; Ns, *P* > 0.05; *P ≤ 0.05; **P ≤ 0.01; ***P ≤ 0.001; ****P ≤ 0.0001

In order to test the relevance of the identified Bet_v_1 ligands in processing and presentation by DCs in a time‐dependent manner, we incubated BMDCs with Bet_v_1 in complex with different ligands and detected the presentation of Bet_v_1 by using CD4^+^ T‐cell hybridoma cells specific for the immune‐dominant T‐cell epitope (Thr142‐Ala153). T‐cell proliferation was monitored indirectly by IL‐2 secretion (Figure 5). Interestingly, Bet_v_1 in complex with PPE_1_ consistently affected the MHCII presentation of Bet_v_1 epitope on DCs (Figure 5B‐F), whereas in complex with DOC epitope presentation was affected only after 48 hours (Figure 5F). In contrast, Q3OS and PPB_1_ did not affect the presentation of Bet_v_1.

## 4 DISCUSSION

Th2 polarization cannot be explained by allergenic proteins exclusively; instead, components of the pollen extract significantly contribute to the process of allergic sensitization.^2^ In this context, pollen‐derived compounds able to bind allergens represent promising candidates in the search for additional factors complementing Bet_v_1 allergenicity.^11^, ^35^, ^36^, ^37^, ^38^, ^39^ Structurally, this property is encoded by Bet_v_1’s hydrophobic binding pocket, which can harbor compounds of up to 1400 Da.^40^, ^41^ Here, for the first time, we observed that Bet_v_1 binds phytoprostanes, but not the TLR agonists LTA and LPS. The pollen‐derived ligands Q3OS and PPE_1_, as well as DOC, have micromolar affinities to Bet_v_1, comparable to previously published values for Q3OS and DOC.^22^, ^26^

PPE_1_ inhibits the production of IL‐12p70 in LPS‐stimulated human DCs via blocking of NF‐κB and activation of PPAR‐γ, thus favoring a Th2‐dominated immune response.^23^, ^33^ By contrast, we found that stimulation of moDCs by PPE_1_ in complex with Bet_v_1 without additional LPS‐co‐stimulation did not upregulate maturation markers nor alter cytokine expression, neither did Bet_v_1 alone nor Bet_v_1 in complex with Q3OS or DOC. These discrepancies can be explained by the additional treatment with LPS, which via activation of TLR4 can induce expression of maturation markers.^42^

It has been suggested that diminished proteolytic processing of antigens results in low loading and density of class II MHC‐peptide complexes, thus favoring Th2 polarization.^18^ Our results revealed that ligand binding resulted in an overall protein‐stabilizing effect. Increased thermal stability tended to correlate with proteolytic stability, which in turn affects immunogenicity/allergenicity.^17^ Indeed, the susceptibility of Bet_v_1 to degradation by endolysosomal extracts was substantially reduced by the ligands DOC and PPE_1_. Due to its complexity, the reaction conditions of the endolysosomal fraction cannot be easily controlled, but its degradation pattern can largely be mimicked by cathepsin S, allowing us to establish an in vitro degradation system.^21^ Here, we revealed significant legumain activity as a component of the endolysosomal fraction, albeit with lower fluorescence signal. Consequently, legumain was included in the in vitro degradation system. Importantly, legumain is not a member of the papain‐like protease clan and therefore possesses mechanistic properties, substrate profiles, and inhibition profiles that are fundamentally different from cathepsins.^29^

Investigation using the in vitro degradation system revealed that Bet_v_1 ligands can tune Bet_v_1 endolysosomal processing in two mechanistically different ways. Firstly, ligands affected the allergen processing primarily with respect to the relative abundance of generated peptides available for MHC presentation. Secondly, the newly identified Bet_v_1 ligand PPE_1_ selectively inhibited cathepsin S and other papain‐like cysteine proteases, but not legumain. Why PPE_1_, but not the two structurally related phytoprostanes PPB_1_ and PPF_1_, possesses this inhibitory function can be explained by the chemical structure of PPE_1_, which differs from PPB_1_ and PPF_1_ at the five‐membered ring^43^ (Figure 4C). The mechanistic explanation for the cathepsin S‐inhibitory effect is that, under acidic conditions, PPE_1_ can spontaneously undergo dehydration,^43^ converting the five‐membered ring into an electrophilic Michael acceptor. The cyclopentenone favors the addition of the nucleophilic thiolate of the catalytic cysteine, thereby covalently blocking the protease active site (Figure 4E). The access to the active site of legumain is sterically more stringently controlled than the active site of papain‐like proteases,^32^ explaining why legumain neither reacts with nor is inhibited by PPE_1_. The reactive 3‐hydroxy‐cyclopentanone is commonly found in plants^44^ and, in particular, was identified in birch pollen.^9^, ^45^ PPE_1_ was found in plants at concentrations ranging from 4.5 to 61 ng per gram of dry weight.^44^

The immunological relevance of these unexpected findings was even demonstrated in a T‐cell proliferation assay, showing a unique reduction in the presentation of the T‐cell epitopes when Bet_v_1 was complexed with PPE_1_. This drastic effect can mostly be explained by PPE_1_’s cysteine cathepsin‐inhibition function, and hardly to its stabilizing properties since such an effect was not observed for PPB_1_.

So far, it is unknown whether Bet_v_1 homologues from other pollen or food sources are able to bind ligands, which enables them to further increase their allergenicity in terms of proteolytic stability, processing, T‐cell proliferations, or IgE binding. Especially, in the light of the pollen‐food syndrome, future studies investigating ligand binding of clinically relevant Bet_v_1 homologues, such as Cor a 1, are required.^46^

To summarize, we identified an unexpected mechanism by which Bet_v_1 serves as a carrier of an endosomal inhibitor, which interferes with the main class of antigen‐processing proteases. Increased proteolytic resistance of Bet_v_1 drastically affects its allergenicity and immunogenicity.^17^ Furthermore, such broad‐spectrum inhibition is likely to change not only the presented immunopeptidome but also the proteolytic activation of endosomal and intracellular immune receptors like TLRs and NLRs. Additionally, there may be a direct interaction of Bet_v_1 ligands with these receptors.^47^ The relevance of such direct or indirect activation by pollen‐derived non‐proteinogenic molecules can help to reconcile the intriguing finding that the sensitization process by birch pollen extracts is independent from Bet_v_1.^2^

## CONFLICTS OF INTEREST

F. Ferreira is a member of Scientific Advisory Boards (HAL Allergy, NL; SIAF, Davos, CH; AllergenOnline, USA). The remaining authors declare that they have no relevant conflicts of interest.

## AUTHOR CONTRIBUTIONS

WTS, LA, SH, SG, TS, PT, PB, and GM performed the experiments. WTS, LA, GM, SG, R.L, C.T‐H., CC, HB, and FF devised the experiments and interpreted the data. WTS, LA, HB, and F.F wrote the manuscript.
