# Supplementary figures and images for: Multiple roles of Bet v 1 ligands in allergen stabilization and modulation of endosomal protease activity
Source: Allergy. 2019 Oct 8;74(12):2382–93. doi: 10.1111/all.13948 (PMC6910946; doi:10.1111/all.13948)

Fig. S1

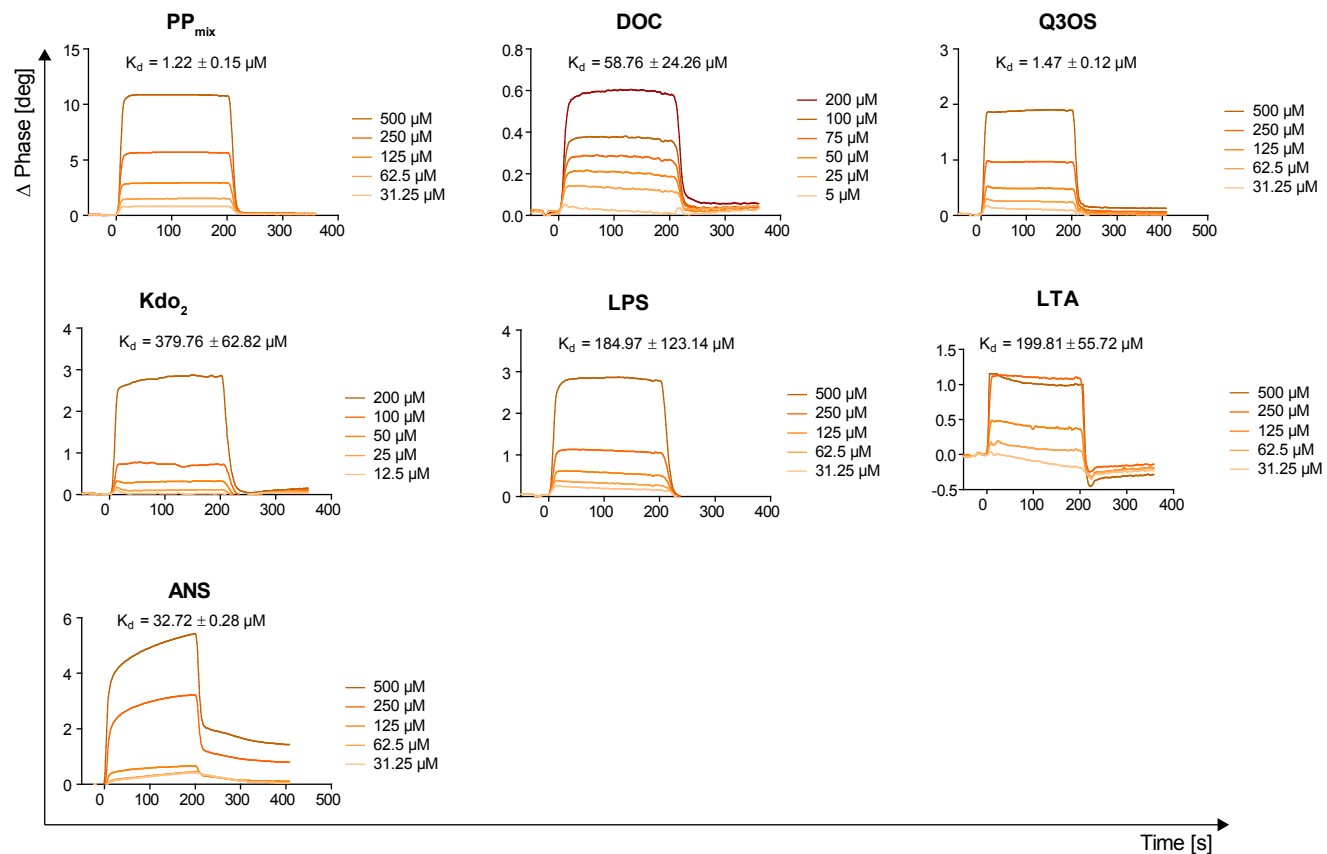

Fig. S2

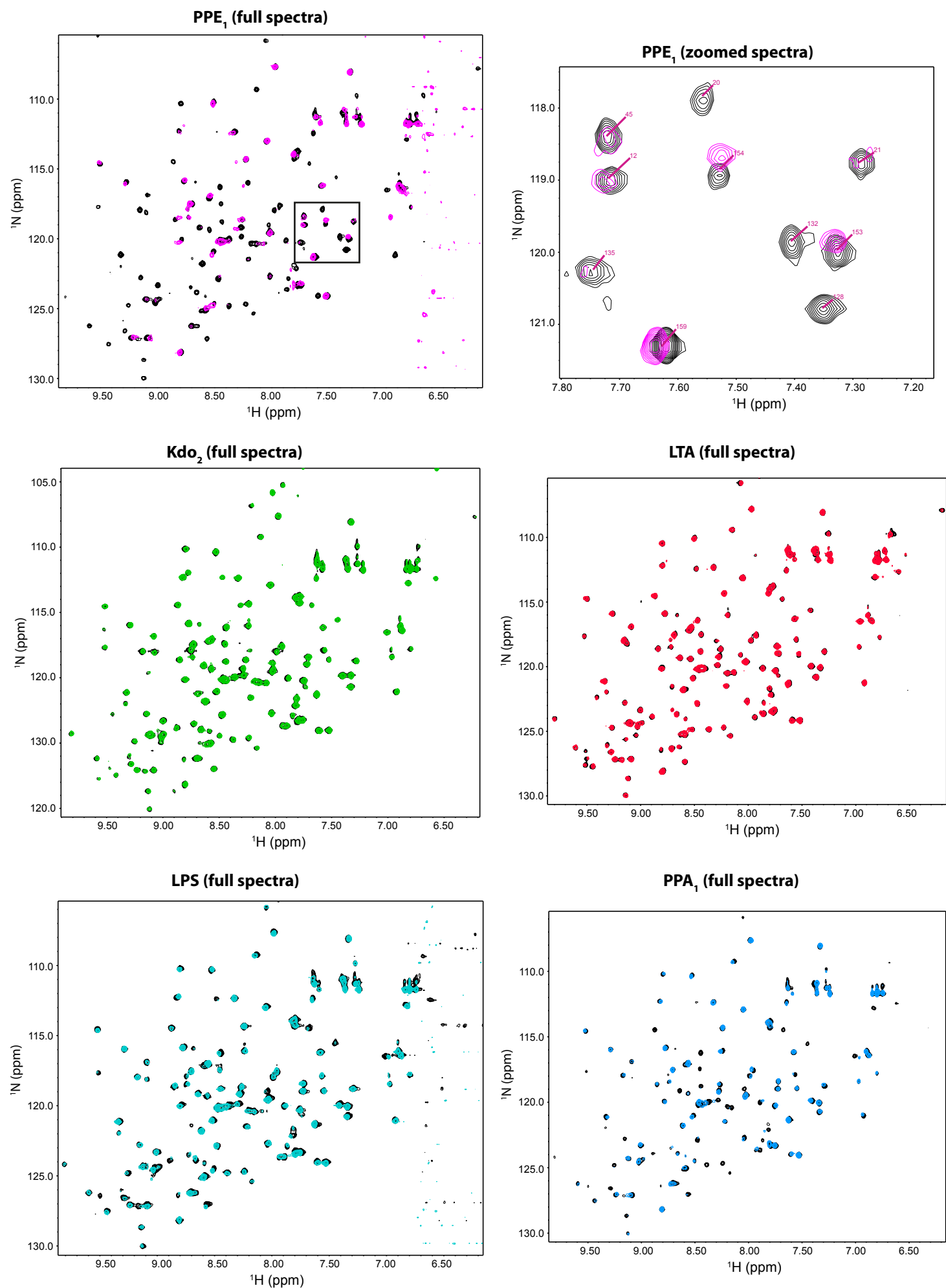

**Fig. S3**

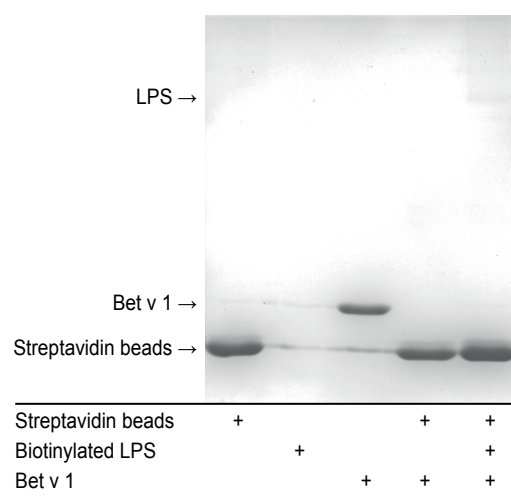

**Fig. S4**

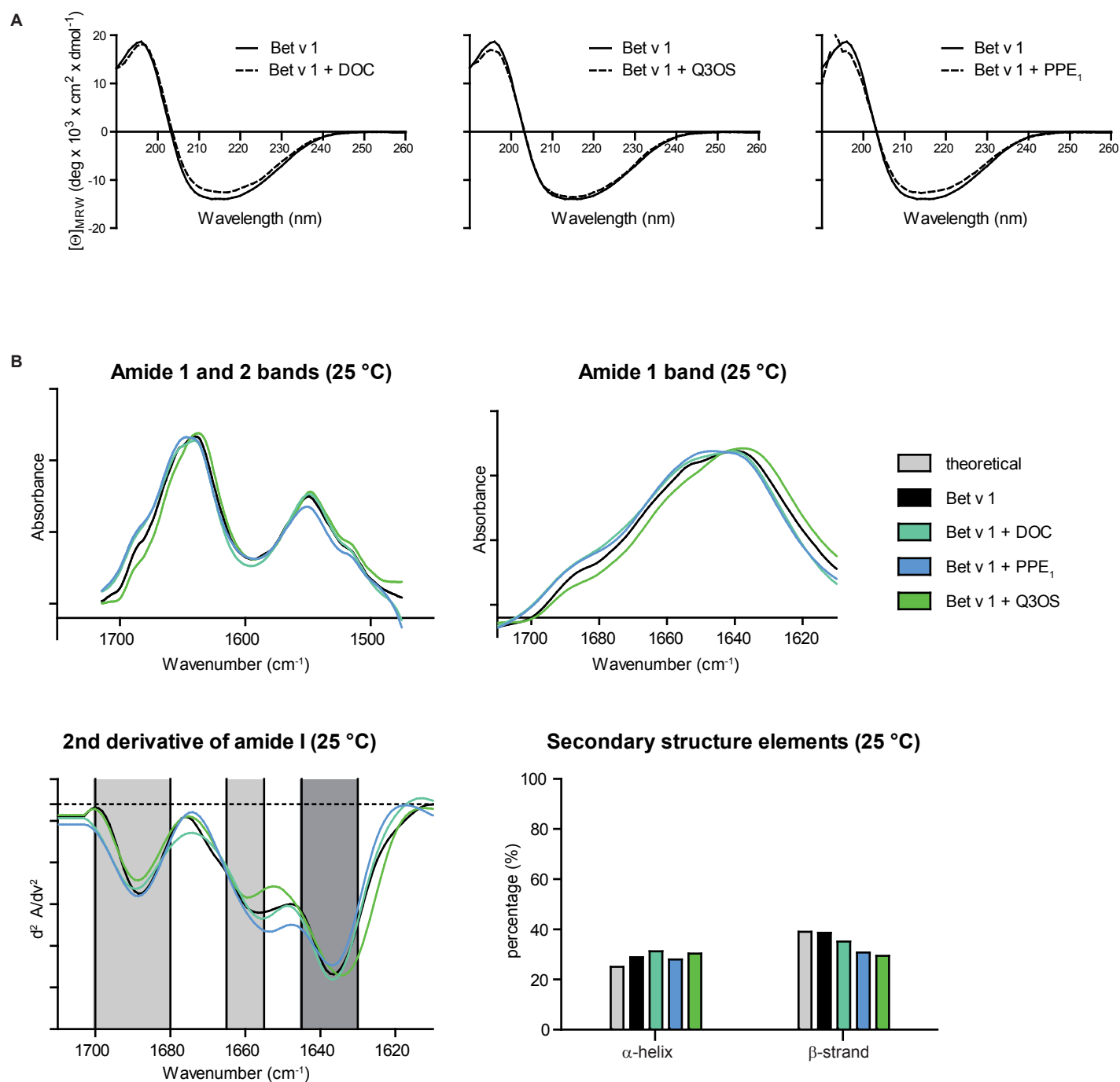

Fig. S5

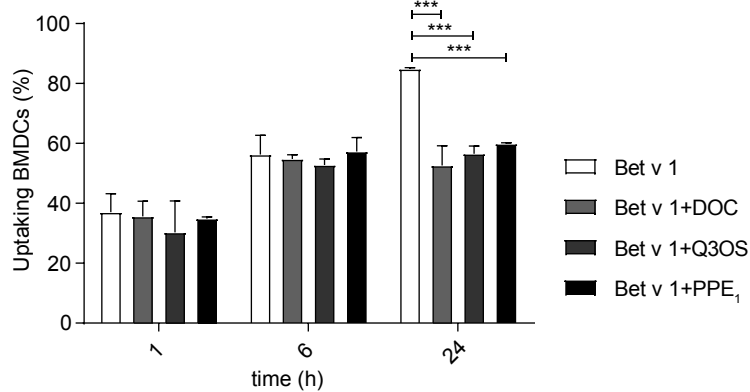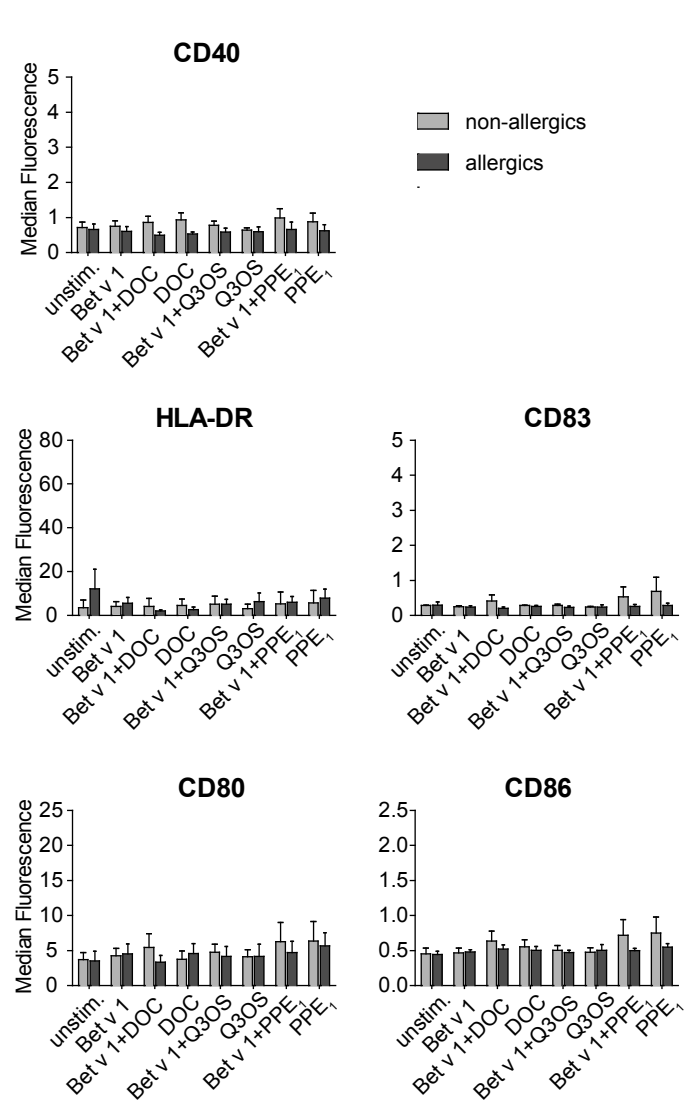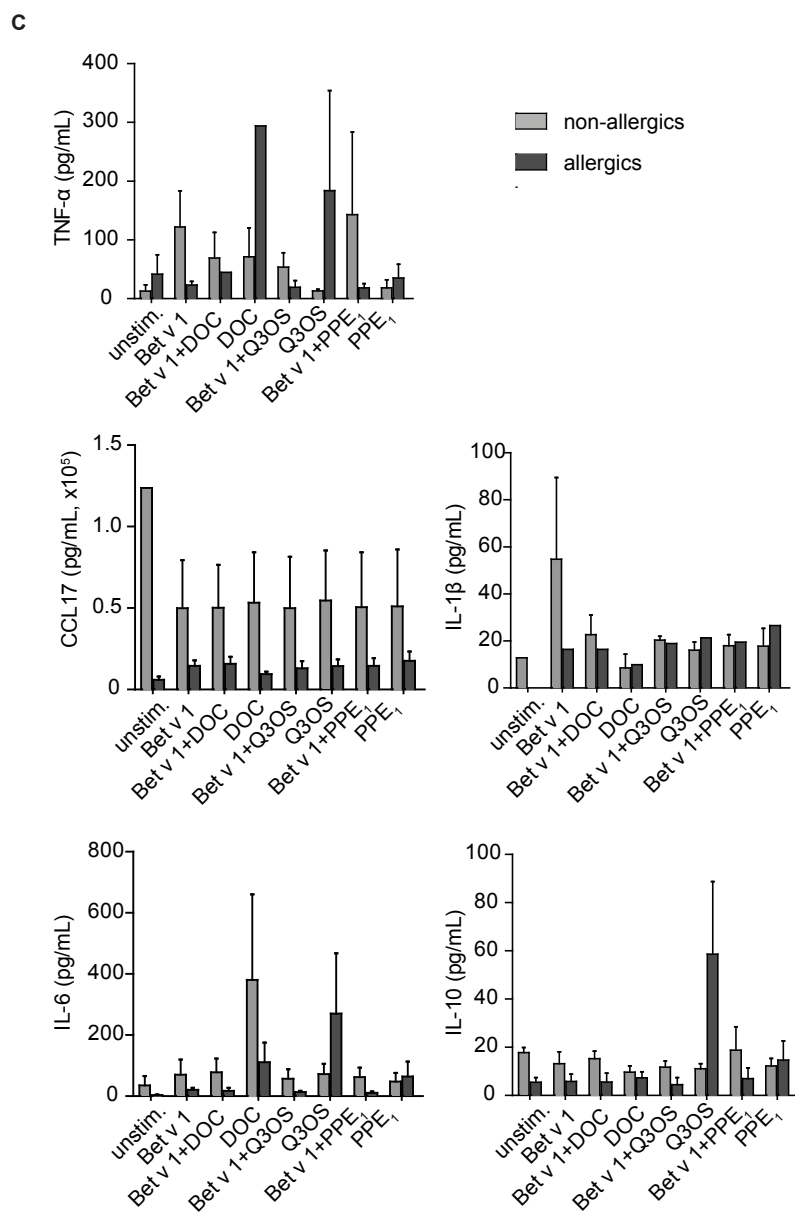

A

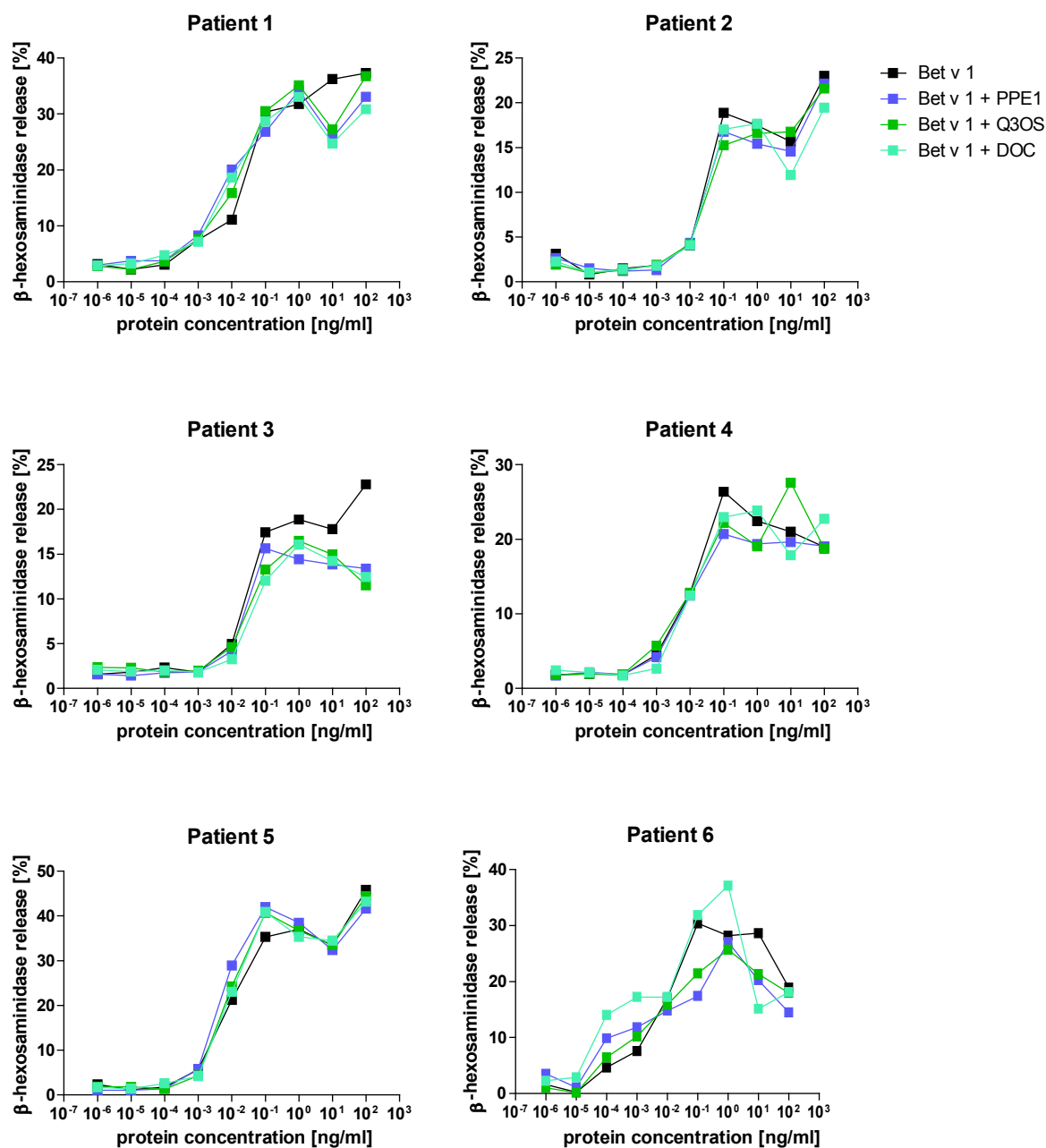

B

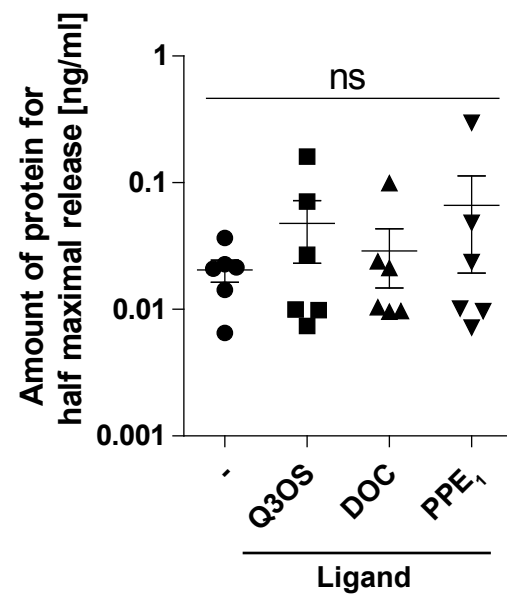

Fig. S7

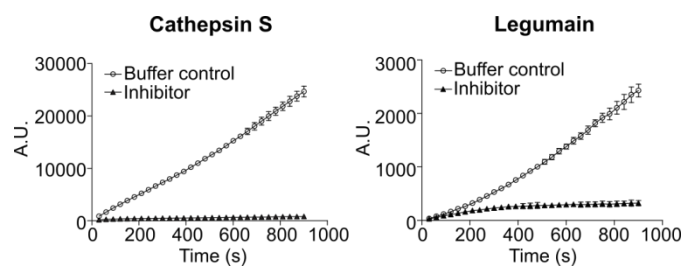

Fig. S8

A.

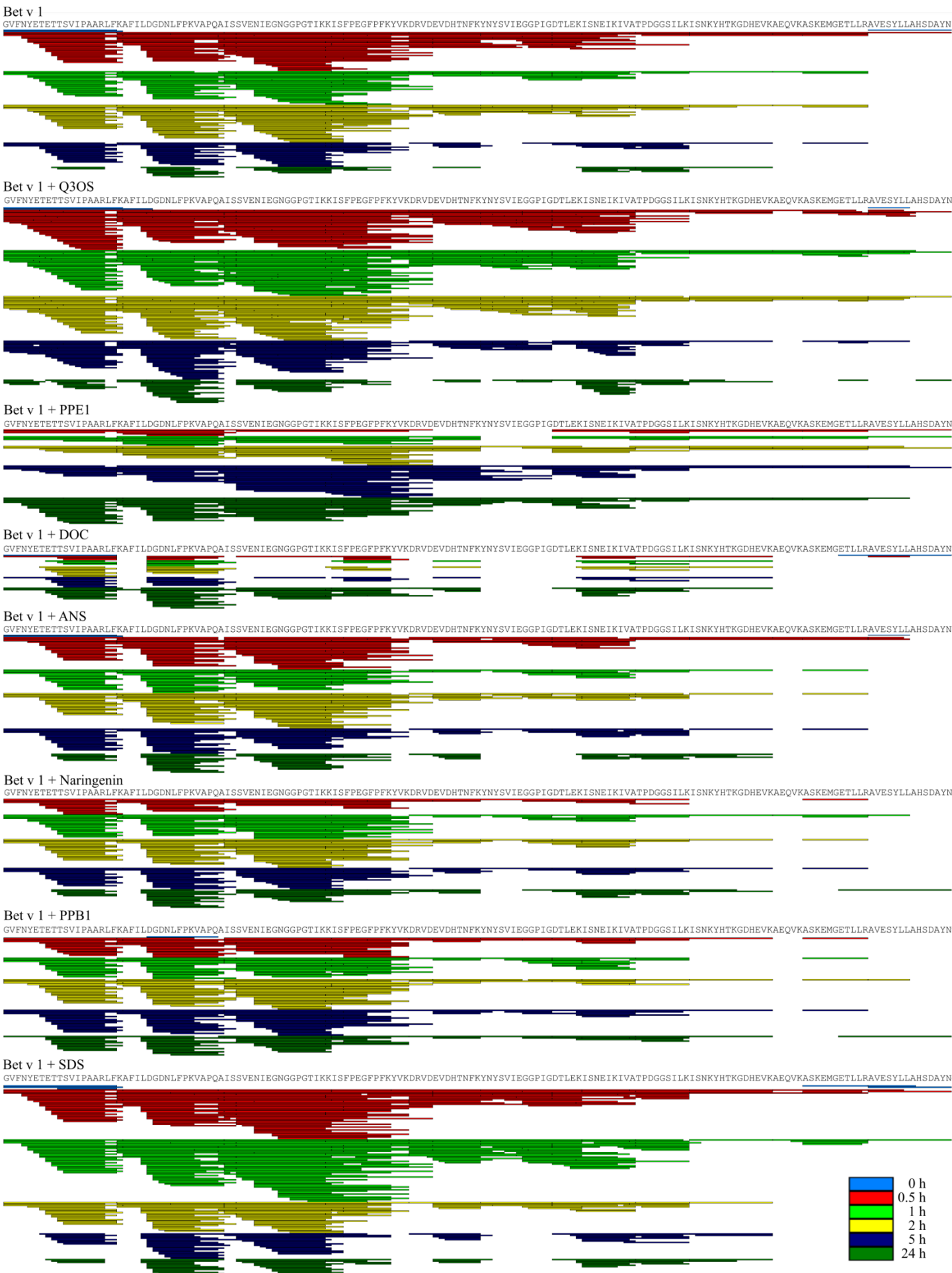

Fig. S8

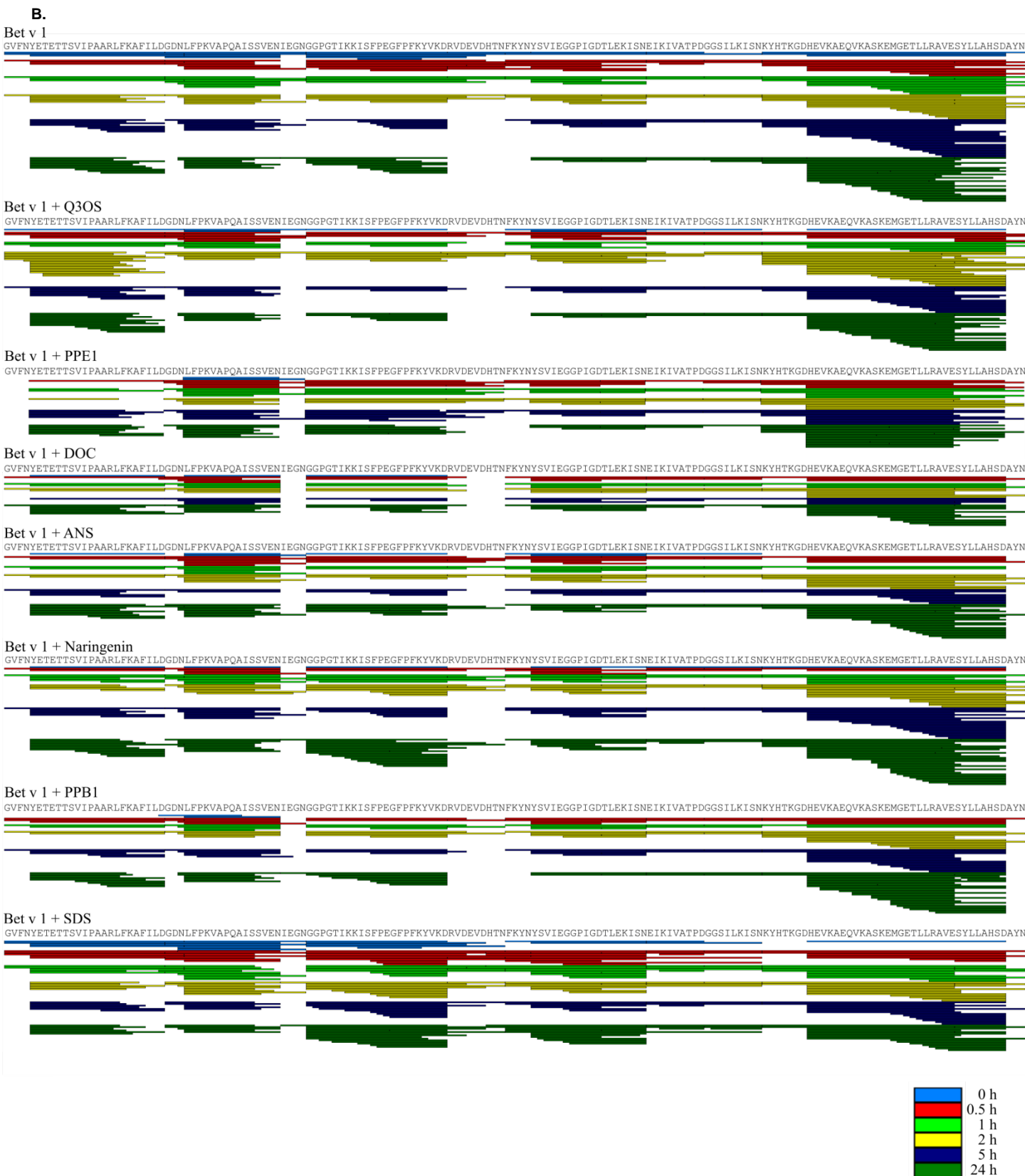

Fig. S9

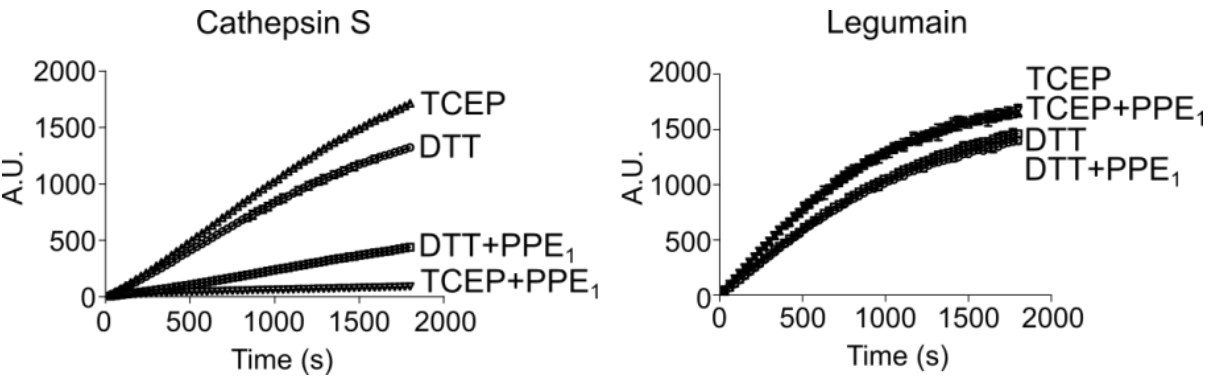

Supplement: Supplementary file 1 [file ALL-74-2382-s001.pdf]
