# Supplementary material for: Multiple roles of Bet v 1 ligands in allergen stabilization and modulation of endosomal protease activity
Source: Allergy. 2019 Oct 8;74(12):2382–93. doi: 10.1111/all.13948 (PMC6910946; doi:10.1111/all.13948)
Supplement: Supplementary file 2 [file ALL-74-2382-s002.doc]

**SUPPORTING INFORMATION**

**Title: MULTIPLE ROLES OF BET V 1 LIGANDS IN ALLERGEN STABILIZATION AND MODULATION OF ENDOSOMAL PROTEASE ACTIVITY**

**Running Title: MULTIPLE ROLES OF BET V 1 LIGANDS**

Wai Tuck SOH MSc ^#a^, Lorenz AGLAS PhD ^#a^, Geoffrey A. MUELLER PhD ^b^, Stefanie GILLES PhD ^c, d^, Richard WEISS PhD ^a^, Sandra SCHEIBLHOFER PhD ^a^, Sara HUBER MSc ^a^, Tamara SCHEIDT MSc ^a^, Peter M. THOMPSON PhD ^b^, Peter BRIZA PhD ^a^, Robert E. LONDON PhD ^b^, Claudia TRAIDL-HOFFMANN PhD ^c, d^, Chiara CABRELE PhD ^a^, Hans BRANDSTETTER PhD ^a *^, Fatima FERREIRA PhD ^a *^

^a^ Department of Biosciences, University of Salzburg, Salzburg, Austria.

^b^ Genome Integrity and Structural Biology Laboratory, National Institute of Environmental Health Sciences, National Institutes of Health, Department of Health and Human Services, Research Triangle Park, NC

^c^ Chair and Institute of Environmental Medicine, UNIKA-T, Technical University Munich and Helmholtz Zentrum München, Augsburg, Germany

^d^ Christine-Kühne-Center for Allergy Research and Education (CK CARE), Davos, Switzerland

^#^ These authors contributed equally to this work.

*** Corresponding authors**

Department of Biosciences, University of Salzburg

Hellbrunner Str. 34, A-5020 Salzburg, Austria

Tel.: +43-662-8044-5016, Fax: +43-662-8044-745016

Email: fatima.ferreira@sbg.ac.at

**Supplementary Materials and Methods**

**Allergic Patients**

The sera for mediator-release assays were collected from birch pollen-allergic patients (n = 6) and selected based on case history, positive skin prick test and allergen-speciﬁc IgE reactivity. Detection of total and allergen-specific IgE levels was performed using Immuno-CAP (Thermo Fisher Scientiﬁc, Uppsala, Sweden). The study was approved by the ethics committee of the medical faculty of Technical University of Munich (19/15 S).

**Surface acoustic wave (SAW) interaction studies and determination of the affinity constant K_d_**

For the determination of the affinity constant K_d_, we used surface acoustic wave (SAW) technology. The interaction of the six ligands with Bet v 1 was investigated using a sam® 5BLUE biosensor instrument (Nanotemper, Munich, Germany). Bet v 1 was immobilized in PBS on the surface of a SAW CM-Dextran 3D sensor chip. The surface of the chip was activated with a freshly prepared mixture of NHS (100 mM) and EDC (400 mM), and free activated sites were subsequently blocked with ethanolamine, pH 8.5 (1 M). For calculating the affinity constant K_d_, different concentrations of the ligands dissolved in a 5 mM sodium phosphate buffer, pH 7.4, were applied to the Bet v 1-coupled chip. Depending on the ligand, the concentration range of the injected ligand varied between 5 µM and 500 µM. For the removal of residual ligand between the sample injections, the chip was washed with regeneration buffer (10 mM citric acid buffer, pH 2.8). The recorded SAW phase changes were analyzed using the software TraceDrawer 1.7 (Ridgeview Instruments, Uppsala, Sweden). The affinity constant K_d_ was calculated by kinetic evaluation and affinity/EC50 estimation. For kinetic evaluation, an OneToOne curve fit was chosen to match the raw data. Experiments were performed with two coated chips in duplicate, and the coupling efficiency was evaluated using a mouse monoclonal anti-Bet v 1 antibody.

**Expression and Purification of ^15^N-^1^H labeled Bet v 1**

^15^N-^1^H labeled Bet v 1 was expressed following variation of a previously described method.^1^ Two liters of bacteria were grown overnight in LB medium, centrifuged and resuspended in 0.5 L of M9 salts lacking any carbon or nitrogen source and equilibrated for 35 min. Cells, fed with glucose, ^15^N ammonium chloride, and ^15^N Celltone (1/25 g) (Cambridge Isotope Labs), were incubated for 35 min and then induced with IPTG. The protein suspension was purified as described above. In addition, an extra Superdex 75 size-exclusion column (GE Healthcare Biosciences, Little Chalfont, UK) step was used.

**NMR spectroscopy**

The NMR data were acquired with either a 600 or 800 MHz Agilent DD2 spectrometer with a cryogenically cooled probe using the pulse sequence gNhsqc. Bet v 1 assignments were taken from.^2^ The experiments were performed under the following conditions: LTA: 45 µM Bet v 1 and 300 µM LTA in PBS, 10% ^2^H_2_O, 60 µM DSS; PPE_1_: 25 µM Bet v 1 and 2 µM PPE_1_ in PBS, 5% ^2^H-(d6) DMSO, 10% ^2^H_2_O, 60 µM DSS; PPA_1_: 100 µM Bet v 1 and 302 µM PPA_1_ (Cayman Chemical, MI, USA) in PBS, 8.7% ^2^H-(d6) DMSO, 10% ^2^H_2_O, 60 µM DSS; LPS: 100 µM Bet v 1 and 400 µM LPS in PBS pH 7.4, 10% ^2^H_2_O, 60 µM DSS. Kdo2: 100 µM Bet v 1 and 422 µM Kdo2 in PBS pH 7.0, 10% ^2^H_2_O, 8.6% ^2^H-(d6) DMSO, 60 µM DSS.

**LPS pull-down assay**

To investigate whether Bet v 1 binds LPS, a pull-down assay using biotinylated ultrapure *E. coli* O111:B4 LPS (InvivoGen, San Diego, CA, USA) was performed as previously described.^3^ In short, biotinylated LPS was immobilized on Strep-Tactin Sepharose beads (IBA Lifesciences, Göttingen, Germany) and incubated with Bet v 1 for 20 min at room temperature. Unbound protein was washed off and beads were analyzed by SDS-PAGE. As control, the individual components (Bet v 1, biotinylated LPS and Strep-Tactin Sepharose beads), and Bet v 1+beads to exclude unspecific binding, were analyzed.

**Determination of secondary structure elements and thermal stability**

The influence of ligand binding on the secondary structure elements and the thermal stability of Bet v 1 was addressed by circular dichroism (CD) and Fourier transform infrared (FTIR) spectroscopy. CD spectroscopy was performed with a JASCO J-815 spectropolarimeter fitted with a PTC-423S Peltier-type single position cell holder (Jasco, Tokyo, Japan) over the wavelength range 190 to 260 nm. For measurement, Bet v 1 with and without ligand was diluted in a 10 mM potassium phosphate buffer to a concentration of 0.1 mg/ml. The thermal stability analysis was recorded at 222 nm. The protein was denatured with a temperature slope of 1°C/min from 20 to 95°C.

FTIR amide 1 and amide 2 spectra of Bet v 1 at concentrations of 1.0-2.0 mg/ml with and without ligand were recorded with an AquaSpec transmission cell adapted to a Tensor II FTIR system (Bruker Optics Inc., Billerica, MA, USA). Measurement was performed at a constant temperature (25°C), controlled by a Haake F8 thermostat (Thermo Electron, Germany) with internal temperature control. For data analysis, OPUS spectroscopy software 6.0 (Bruker Optics Inc., Billerica, MA, USA) was used. The Savitzky-Golay algorithm was applied on vector-normalized amide 1 spectra (25 smoothing points) to obtain the second derivative. For the quantification of secondary structure elements, a Quant2 method of the OPUS software was used. For thermal stability analysis, spectra were recorded with a BioATR II unit (Bruker Optics Inc., Billerica, MA, USA) at 25 to 95°C (dT=2.5K). The determined relative signal change in the amide I band by creating a difference spectrum for each temperature point was used to calculate the melting point (Tm).

**Mediator release assay**

A mediator-release assay was performed to assess the capacity of ligand-loaded Bet v 1 to induce IgE-antigen-crosslinking and basophil degranulation. In this respect, rat basophil (RBL-2H3) cells, transfected with the human high-affinity IgE receptor (FcεRI) were passively sensitized with sera of six subjects allergic to birch pollen (1:10), and used as previously described.^4^ For cell-stimulation, Bet v 1 was used at 10-fold dilutions from 1 µg/ml to 0.01 pg/ml. Toxicity of ligands on RBL-2H3 was determined by cell viability assay using MTT (3-(4,5-Dimethylthiazol-2-yl)-2,5-Diphenyltetrazolium Bromide) from Sigma-Aldrich, Inc. (St. Louis, MO, USA). Results were expressed as percentage of total enzyme release of cells lysed with Triton X-100, and the half maximal release (in ng) was determined. Statistics were calculated with a repeated ANOVA followed by a Dunnett post-hoc test of logarithmically transformed half maximal release values (not significant, ns P > 0.05, *P < 0.05, **P < 0.01, and ***P < 0.001).

***In vitro* antigen uptake of murine bone marrow-derived dendritic cells (BMDCs)**

BMDCs from C57BL/6 mouse bone marrow were isolated as described previously.^5^ cultured in BMDC medium (RPMI 1640, 5% fetal calf serum, 2 mM L-glutamine, 1% penicillin-streptomycin, 20% granulocyte-macrophage colony-stimulating factor supernatant and 200 µM β-mercaptoethanol) (BMDC medium) for 10 days, and stored frozen in liquid nitrogen. BMDCs were treated over a certain period of time (24, 6 or 1 hours) with 0.5 µg of Bet v 1 per 2x10^5^ cells. Bet v 1 was labeled with pHrodo™ Red succinimidyl ester (Thermo Fisher Scientific, Waltham, MA, USA) and pre-incubated with one of the ligands (DOC, Q3OS or PPE_1_). CD11c^+^ cells were analyzed via flow cytometry (allophycocyanin-conjugated anti-mouse CD11c antibody, clone N418; eBioscience, Inc., San Diego, CA, USA) on a FACSCanto II instrument (BD Biosciences, San Jose, CA, USA). For the live/dead staining a fixable viability stain 450 (BD Biosciences) was used. Granulocytes and monocytes were excluded via the V450 Rat anti-Mouse LY-6G and LY-6C (BD Biosciences) antibody. Data were recorded and analyzed using the BD FACSDiva software (BD Biosciences). Statistical analysis was performed using ANOVA with a Bonferroni post-test to compare all groups. The data represent the mean of duplicate experiments and are derived from at least 2 independent experiments.

**Activation and cytokine secretion of human monocyte-derived dendritic cells (moDCs)**

Immature moDCs from PBMCs of either healthy/non-atopic donors (n=5) or atopic patients (n=7) were isolated and cultured as described elsewhere.^6^ Cell viability using the Aqua® dye (Invitrogen, Carlsbad, CA, USA), expression of CD1a (eBioscience, Inc., San Diego, CA, USA) and loss of CD14 (BD Biosciences) expression were analyzed by flow cytometry on a Navios flow cytometer (Beckman Coulter, Brea, CA, USA). Cells (1x10^6^ cells/ml) were stimulated for 24 hours with 1 µg/ml of Bet v 1 either alone or in complex with one of the three ligands (DOC, PPE_1_ or Q3OS). As a control, unstimulated moDCs and the individual ligands without Bet v 1 were used at the same concentrations. The cell supernatant was frozen for the analysis of cytokine secretion. Of note, no LPS co-stimulation was induced. The following maturation markers were investigated: CD40 (eBioscience), HLA-DR, CD80, CD83 and CD86 (BD Biosciences). Cytokine expression of CCL17, IL-1β, IL-10 (BD Biosciences), TNFα and IL-6 (eBioscience) was determined by ELISA. Statistical analysis was performed using one-way ANOVA with a Bonferroni post-test to compare all groups. The study was approved by the ethics committee of the medical faculty of Technical University of Munich (54/17 S).

***In vitro* simulation of endolysosomal degradation**

The endolysosomal degradation assay was performed with ligand-bound (either DOC, PPE_1_, or Q3OS in 10× molar excess) and Bet v 1 without ligands (apo-Bet v 1) as previously described.^7^ The protein (5 µg) was incubated with 7 µg of microsomes in 100 mM citrate buffer pH 4.8 and 2 mM dithiothreitol (DTT) for 0, 0.5, 1, 3, 6, 12, 24, and 48 h at 37°C. The different digestion preparations were analyzed by SDS-PAGE and quantitatively interpreted using Image Lab 4.0.1 Software (Bio-Rad). The generated peptides obtained after 12 h of digestion were analyzed using mass spectrometry with a Q-Exactive Orbitrap Mass Spectrometer (Thermo Fisher Scientific, Waltham, MA, USA), nanoelectrospray, and nano-HPLC (Dionex Ultimate 3000, Thermo Fisher Scientific).

**Data analysis of *in vitro* simulation of endolysosomal degradation**

For data analysis, the web-based application MSTools was used.^8^ For the semi-quantitative analysis of the abundance of the generated peptides at 12 hours of degradation, the obtained peptide sequences were clustered into 7 groups according to their amino acid sequence (cluster 1, GVF-NYE-TET-TSV-IPA-ARL-FKA-F; cluster 2, AIL-DGD-NLF-PKV-APQ-A; cluster 3, AIS-SVE-NIE-GNG-GPG-TIK-KIS-FPE-G; cluster 4, FPF-KYV-KDR-VDE-VDH-TNF-K; cluster 5, YNY-SVI-EGG-PIG-DTL-EKI-SNE-IKI-VAT-PDG-GSI-LK; cluster 6, ISN-KYH-TKG-DHE-VKA-EQV-KAS-KEM-GET-LLR; and cluster 7, RAV-ESY-LLA-HSD-AYN), and the total sum of peak areas of all sequences per cluster was calculated (in percentage).

**Bet v 1 *in vitro* proteolytic degradation assay using individual endolysosomal proteases**

Recombinant human cathepsin S and human legumain were heterologously expressed and purified as described previously.^9,10^ Bet v 1 (20 µM) was incubated with ligand (100 µM) in digestion buffer (0.1 M sodium acetate, pH 5.0, 0.1 M sodium chloride, 5 mM EDTA, and 2 mM DTT) at room temperature prior to the addition of cathepsin S (1 µM) or legumain (2 µM). The reactions were incubated at 37°C for 0, 0.5, 1, 2, 5, and 24 h. At each time point, the reaction was halted by the addition of E64 (for cathepsin S) or YVAD-CMK (for legumain) at a final concentration of 50 µM, and analyzed by SDS-PAGE. Control reactions without the addition of protease were performed. The generated peptides were analyzed using mass spectrometry.

**Enzymatic activity assays**

For the detection of cathepsin or legumain activities in the microsomal fraction, 8 µg of microsomal extract was incubated with 50 µM fluorogenic substrate with or without inhibitor in assay buffer (100 mM citrate pH 4.8 and 2 mM DTT). The enzymatic reactions were carried out in a 50 µL reaction volume in a 96-half-well black flat bottom assay plate (Corning Inc., Corning, NY, USA) at 37°C. The fluorescence signal was recorded using a Tecan Infinite-200 plate reader (Tecan, Männedorf, Switzerland) at an excitation wavelength of 380 nm and emission wavelength of 460 nm for 15 min of continuous measurement with a 30-s interval reading. The fluorogenic substrates used for cathepsin S and legumain were Z-VVR-AMC and Z-AAN-AMC (Bachem, Bubendorf, Switzerland), respectively. The inhibitors used for cathepsin S and legumain were Z-FL-COCHO (Sigma-Aldrich, CA, USA) and Ac-YVAD-CMK (Bachem), respectively^11^.

**Preparation of aqueous birch pollen extract (BPE)**

Pollen extract was prepared by incubating 250 mg of Birch pollens (Batch: 012517101, Allergon AB, Ängelholm, Sweden) in 1 mL of extraction buffer (10 mM Tris pH 7.5, 150 mM NaCl) at 4°C under vigorous shaking conditions for 22 hours. Extract was recovered by centrifugation at 12000 g, 4°C for 30 minutes and subsequently filtered through a 0.22 µm filter (Pall, Dreieich, Germany) which was prewashed with extraction buffer twice. Protein concentration was estimated using Bradford protein assay (Bio-Rad, Vienna, USA) using BSA (bovine serum albumin) as standard.

**Mass spectrometry**

Samples were desalted using PierceC18 Tips (Thermo Fisher Scientific, Bremen, Germany) and injected into an UltiMate 3000 RSLCnano HPLC system (Thermo Fisher Scientific, Waltham, MA, USA) by using a 1.0 µL-Pickup injection. At a flow rate of 350 nL/min, peptides were separated using an in-house packed 200 x 0.1 mm i.d. Hypersil GOLD™ aQ C18 capillary column with 3.0 µm particles. Water (A) and acetonitrile (B) with 0.10 % (v/v) formic acid (FA, Sigma Aldrich) were used as eluents. For the separation, the column temperature was set to 50.0°C and a linear gradient of 5.0-40.0% B in 100.0 min was used. The HPLC-system was hyphenated to a Q Exactive™ Hybrid Quadrupole-Orbitrap™ mass spectrometer (Thermo Scientific) by using a nano-electrospray ionization source. The source was operated in positive ion mode with a spray voltage of 1.5 kV. Ionized peptides were measured using data dependent acquisition based on the Top 15 most abundant precursor ions with a full MS resolution of 70,000, AGC target of 1e6, maximum injection time of 120 ms with a scan range of 370 to 2000 m/z. Fragment spectra were recorded with a resolution of 35,000 applying a normalized collision energy (NCE) of 29. The AGC target was set to 2e5 with a maximum injection time of 100 ms and an isolation window of 2.0 m/z. The dynamic exclusion was set to 45.0 s.

Survey and fragment spectra were analyzed with PEAKS Studio 8 (Bioinformatics Solutions, Waterloo, ON, Canada). Searches were done with a Bet v 1 database. Only peptides with a high confidence score (-10lgP ≥ 35) were considered for further analysis.

**T-cell proliferation assay**

Bet v 1 was incubated over night with a 10-fold molar excess of either Q3OS, PPB_1_, PPE_1_, or DOC before incubation with murine BMDCs at a concentration of 10 μg/mL in BMDC medium (online supporting information). After 16, 24, 32, and 48h, DCs were washed and CD4^+^ T-cell hybridomas specific for the immune-dominant epitope 142-153 of Bet v 1 were added to the culture at a ratio of 1:10 (DC:T-cell) for 24h^17^. Supernatants were harvested and IL-2 levels were determined by ELISA (Invitrogen, Thermo Fisher Scientific, Waltham, MA, USA). As a reference, BMDCs were pulsed at each time point with different concentrations of peptide 142-153 and co-cultured with CD4^+^ T-cell hybridomas. By interpolating in a standard curve, a µM peptide equivalent from the measured IL-2 was calculated from DCs pulsed with different peptide concentrations. The assays were carried out in triplicates.

**References**

1. Marley J, Lu M, Bracken C. A method for efficient isotopic labeling of recombinant proteins. *J Biomol NMR.* 2001;20(1):71-75.

2. Grutsch S, Fuchs JE, Ahammer L, Kamenik AS, Liedl KR, Tollinger M. Conformational Flexibility Differentiates Naturally Occurring Bet v 1 Isoforms. *Int J Mol Sci.* 2017;18(6).

3. Herre J, Gronlund H, Brooks H, et al. Allergens as immunomodulatory proteins: the cat dander protein Fel d 1 enhances TLR activation by lipid ligands. *J Immunol.* 2013;191(4):1529-1535.

4. Nowak-Wegrzyn AH, Bencharitiwong R, Schwarz J, et al. Mediator release assay for assessment of biological potency of German cockroach allergen extracts. *J Allergy Clin Immunol.* 2009;123(4):949-955 e941.

5. Behboudi S, Chao D, Klenerman P, Austyn J. The effects of DNA containing CpG motif on dendritic cells. *Immunology.* 2000;99(3):361-366.

6. Gilles S, Mariani V, Bryce M, et al. Pollen-derived E1-phytoprostanes signal via PPAR-gamma and NF-kappaB-dependent mechanisms. *J Immunol.* 2009;182(11):6653-6658.

7. Egger M, Jurets A, Wallner M, et al. Assessing Protein Immunogenicity with a Dendritic Cell Line-Derived Endolysosomal Degradome. *Plos One.* 2011;6(2).

8. Kavan D, Man P. MSTools—Web based application for visualization and presentation of HXMS data. *International Journal of Mass Spectrometry.* 2011;302(1–3):53-58.

9. Freier R, Dall E, Brandstetter H. Protease recognition sites in Bet v 1a are cryptic, explaining its slow processing relevant to its allergenicity. *Sci Rep-Uk.* 2015;5.

10. Dall E, Brandstetter H. Mechanistic and structural studies on legumain explain its zymogenicity, distinct activation pathways, and regulation. *P Natl Acad Sci USA.* 2013;110(27):10940-10945.

11. Wildner S, Elsasser B, Stemeseder T, et al. Endolysosomal Degradation of Allergenic Ole e 1-Like Proteins: Analysis of Proteolytic Cleavage Sites Revealing T Cell Epitope-Containing Peptides. *Int J Mol Sci.* 2017;18(8).
